# Melatonin Alleviates LPS-Induced Pyroptotic Cell Death in Human Stem Cell-Derived Cardiomyocytes by Activating Autophagy

**DOI:** 10.1155/2021/8120403

**Published:** 2021-11-27

**Authors:** Ya Qiu, Yan Ma, Min Jiang, Sulei Li, Jibin Zhang, Haixu Chen, Mengqi Xu, Shan Gao, Lei Tian, Bo Tao, Yabin Wang, Dong Han, Feng Cao

**Affiliations:** ^1^Institute of Geriatrics, Beijing Key Laboratory of Aging and Geriatrics, National Clinical Research Center for Geriatrics Diseases, 2nd Medical Center of Chinese PLA General Hospital, 100853 Beijing, China; ^2^National Clinical Research Center for Geriatric Diseases & 2nd Medical Center, Chinese PLA General Hospital, 100853 Beijing, China

## Abstract

Endotoxemia in sepsis remains a problem due to a lack of effective strategies. Our previous studies have demonstrated that melatonin (Mel) protects against ischemic heart injury and arteriosclerosis. However, its role in endotoxemia-exposed cardiomyocytes remains poorly understood. This study explored, for the first time, the protective effect of Mel on the pyroptosis of human stem cell-derived cardiomyocytes (hiPSC-CMs) exposed to lipopolysaccharide (LPS). Our results showed that treatment with 1 *μ*M or 10 *μ*M Mel for 12 h significantly improved 1 *μ*g/ml LPS-induced hiPSC-CM injuries, as reflected by drastically decreased LDH release and increased cell viability, which was accompanied by the overt induction of autophagy. Specifically, Mel profoundly alleviated LPS-induced cell pyroptosis, as evidenced by decreased propidium iodide (PI) and active caspase-1 double-positive cell rates; suppressed the expression of NLRP3, cleaved caspase-1 (activated form of caspase-1), and GSDMD-NT (functional N-terminal fragment of GSDMD) expression; and inhibited the production of the cleaved IL-1*β* and cleaved IL-18 cytokines. Additionally, double-membrane autophagosomes were observed in LPS-injured hiPSC-CMs treated with 1 *μ*M or 10 *μ*M Mel. The hiPSC-CMs treated with LPS exhibited considerably fewer acidic vesicles (as revealed by LAMP1 staining) and autophagosomes (as revealed by LC3-II staining); however, Mel reversed this outcome in a dose-dependent manner. Furthermore, coincubation with rapamycin (an autophagy activator) or 3-MA (an autophagy inhibitor) accentuated and attenuated the antipyroptotic actions of Mel, respectively. Collectively, our findings demonstrate that Mel shields hiPSC-CMs against pyroptosis during endotoxemia by activating autophagy.

## 1. Introduction

Sepsis is a severe inflammatory response that causes failure of multiple organs [1–3]. Overexposure to lipopolysaccharide (LPS), a major component of the cell wall of gram-negative bacteria, can stimulate nonimmune cells and initiate the inflammatory process in mammals as a key pathogenic component of sepsis, which is known as endotoxemia [4, 5]. Cardiac dysfunction resulting from sepsis may cause significant morbidity and mortality. At the cellular level, cardiomyocytes die primarily from excessive inflammation; however, the underlying mechanism of their death during sepsis has yet to be fully elucidated.

Pyroptosis is recognized as inflammatory caspase- (mainly caspase-1-) dependent programmed cell death, and it is closely associated with the activation of inflammasomes. The NLRP3 inflammasome is widely known and has been identified primarily in monocytes and macrophages; however, recent studies have demonstrated that it is activated in other cell types, including cardiomyocytes [6]. Inflammasome formation can activate caspase-1 and induce pyroptosis in cardiomyocytes. Activated caspase-1 triggers prointerleukin- (IL-) 1*β* or pro-IL-18 maturation into the proinflammatory cytokines IL-1*β* and IL-18 [7, 8] and cleaves gasdermin D (GSDMD) to yield an N-terminal cleavage product called GSDMD-NT, which is the functional N-terminal fragment of GSDMD that can induce pyroptosis by forming plasma membrane pores and promoting caspase-1 activity through cleavage of procaspase-1 [9]. Recent studies have indicated that pyroptosis occurs in cardiovascular diseases, including septic heart injury [7, 10]. However, whether pharmacological modulation of this highly inflammatory form of lytic programmed cell death improves septic heart injury remains unclear.

Previous investigations have reported that autophagy impairment is associated with the development of cardiac abnormalities [11, 12]. Autophagy refers to a self-degradative process that removes unnecessary or dysfunctional components through a lysosome-dependent mechanism. The most thoroughly researched form of autophagy is macroautophagy, and during this process, the cell forms a double-membrane sequestering compartment known as the phagophore, which matures into an autophagosome [13]. Following fusion with an available lysosome, the cargo of the autophagosome is degraded, and eventually, the contents of the vesicle (now called an autolysosome) are degraded and recycled for reuse [14]. Autophagy can be either prosurvival or prodeath depending on the context, and it involves the degradation of cellular components through lysosomal machinery to maintain cellular homeostasis [15, 16]. The balance between cell survival and cell death is often destroyed in human pathologies, such as inflammation. Nevertheless, there is currently little evidence on the roles of autophagy in pyroptotic cardiomyocyte death during sepsis.

Melatonin (N-acetyl-5-methoxytryptamine, Mel) is an indoleamine predominantly secreted by the pineal gland that has been revealed to exert beneficial effects on cardiovascular diseases, including diabetic cardiomyopathy [17], hypertension [18], myocardial ischemia-reperfusion [19], atherosclerosis [20], and heart failure [21]. We previously demonstrated Mel's protective ability against murine atherosclerosis and myocardial ischemic injury with a focus on attenuation of oxidative stress, inflammation, and apoptosis [19, 20, 22]. However, whether Mel has disease-modifying therapeutic potential in LPS-induced pyroptotic cell death remains unclear.

As a terminally differentiated cell type, research on cardiomyocytes is hampered by difficulties in obtaining cardiac tissues and the inability to propagate heart samples in culture. Consequently, most existing investigations have used animal models to mimic human cardiovascular diseases. However, pivotal physiological and biochemical differences exist between animal and human myocardial tissues, particularly between cardiomyocytes. Over the last decade, human-induced pluripotent stem cell-derived cardiomyocytes (hiPSC-CMs) have emerged as an ideal alternative for the *in vitro* modeling of human cardiovascular diseases [23–25]. Therefore, we used a hiPSC-CM model to examine whether Mel functions as an antipyroptotic agent to reverse LPS-induced cardiomyocyte injury in this study.

## 2. Materials and Methods

### 2.1. Cell Culture and Treatment Protocol

Urinary epithelial cell-derived hiPSCs and all cell culture reagents/kits were purchased from the Beijing Cellapy Biological Technology Company in China. hiPSCs were cultured in PSCeasy medium containing 0.5% penicillin/streptomycin, precoated with a Matrigel solution in culture plates, digested with 0.5 mmol/l EDTA, passaged every 3 days at 80% confluence, and subsequently differentiated into CMs using CardioEasy cardiac differentiation kits (CA2004500; Cellapy), including Induction Media I, II, and III. The PSCeasy medium was replaced with Induction Media I and II sequentially every 48 h, and Induction Medium III was used daily until contracting cells were observed on day 9. Then, the media were replaced with a CardioEasy purification medium (CA2005100; Cellapy) for a no-glucose culture for 4 days (Figure S1). The obtained cardiomyocytes were dissociated with a CardioEasy CM dissociation enzyme kit (CA2006100; Cellapy) per the instructions. Digested CMs were cultured in a CardioEasy maintenance medium (CA2015002; Cellapy) for further tests.

To assess Mel's ability to protect LPS-treated hiPSC-CMs, the cells were pretreated with or without Mel (Sigma-Aldrich, USA, dissolved in ethanol first, then prepared with the PSCeasy medium to final concentrations at 0.1, 1, 10, and 100 *μ*mol/l) for 12 h and incubated with 1 *μ*g/ml LPS (Shanghai Leuven Biological Technology) for 24 h. For autophagy intervention studies, hiPSC-CMs were divided as follows: the control group (CTRL group, cells were maintained in PRMI 1640 medium supplemented with B27 only), LPS group (cells were treated with 1 *μ*g/ml LPS for 24 h), LPS+Mel group (cells were pretreated with 1 *μ*mol/l, 10 *μ*mol/l, or 100 *μ*mol/l Mel for 12 h and then stimulated with 1 *μ*g/ml LPS for 24 h), LPS+Mel+rapamycin group (cells were pretreated with 25 nM rapamycin and 10 *μ*mol/l Mel for 12 h and then stimulated with 1 *μ*g/ml LPS for 24 h), and LPS+Mel+3-MA group (cells were pretreated with 5 mM 3-MA and 10 *μ*mol/l Mel for 12 h and then stimulated with 1 *μ*g/ml LPS for 24 h). Additionally, 30 *μ*mol/l chloroquine pretreatment for 12 h was used to assess autophagy flux.

### 2.2. Immunofluorescence Staining

Cells were fixed in 4% formaldehyde at 4°C, washed with PBS twice, permeabilized with 0.1% Triton X-100, and blocked with 3% bovine serum albumin (BSA) at room temperature for 30 mins. hiPSCs were incubated with specific antibodies against Oct4, SSEA4, and Sox2 (1 : 100, Santa Cruz, USA) for 24 h at 4°C, while hiPSC-CMs were incubated with specific antibodies against *α*-actinin and cTnT (1 : 100, Santa Cruz, USA) for 24 h at 4°C. After the respective treatments, the cells were incubated with specific antibodies against LC3-II, LAMP1, and *α*-actinin (1 : 100, Abcam, USA). The cells were rinsed 3 times using PBS and labeled with Alexa Fluor594 or Alexa Fluor488 secondary antibodies (1 : 1000; Invitrogen, USA) for 1 h at 37°C in the dark. Nuclei were labeled with 4,6-diamidino-2-phenylindole (Abcam, USA) for 5-10 minutes. Fluorescence photos were obtained with an Olympus confocal microscope (Tokyo, Japan).

### 2.3. Cell Viability Assay

Cell viability was assessed by a CCK-8 kit (Dojindo, Japan). Ten microliters of CCK-8 solution was added to 5 ~ 6 × 10^4^ hiPSC-CMs in 96-well plates precoated with Matrigel solution, and the mixtures were incubated for 1.5-3 h at room temperature. The absorbance was automatically determined at 450 nm with a microplate reader (TECAN, Switzerland).

### 2.4. Lactate Dehydrogenase Release

A cytotoxicity assay kit (Beyotime, China) was used to measure LDH release following the instructions. Optical density was spectrophotometrically measured at 490 nm.

### 2.5. FACS Analysis

The pyroptotic cell death rate was measured by a FLICA 660 Caspase-1 Assay Kit (Immunochemistry, USA) using flow cytometry analysis (BD, USA). After interventions and digestion, hiPSC-CMs were centrifuged and washed with cold PBS. Per the manufacturer's protocol, a binding buffer and FLICA 660-YVAD-FMK were added to each flow tube, which was kept away from light, at room temperature for 15 minutes. Next, binding buffer and PI dye were added and blended, and the samples were analyzed by flow cytometry.

### 2.6. Cytokine Measurements

Inflammatory factors IL-18 and IL-1*β* were measured with commercially available ELISA kits (R&D Systems, USA). All samples were detected in triplicate to ensure accuracy.

### 2.7. Transmission Electron Microscopy

hiPSC-CMs were washed with precooled PBS and centrifuged at 1000 × g at 37°C for 5 min. The samples were collected and fixed using 0.1 M cacodylate buffer (pH = 7.2) and ice-cold 2.5% glutaraldehyde at 4°C for 1 h, fixed with 1% osmium tetroxide for 1 h, dehydrated, and embedded in Epon. Then, 0.1 *μ*m thin sections were dyed using uranyl acetate and lead citrate. Photos were obtained by a transmission electron microscope (Hitachi, Model H-7650).

### 2.8. Western Blot Analysis

Proteins were extracted from hiPSC-CMs using cell protein extraction kits. Western blotting was conducted with a standard method. Membranes were incubated at 4°C overnight using primary antibodies, including NLRP3, cleaved caspase-1, GSDMD-NT, cleaved IL-1*β*, cleaved IL-18, LC3, p62, LAMP1, and GAPDH (1 : 800 dilution, Cell Signaling Technology, USA), and incubated with specific secondary antibodies (1 : 5000, Proteintech) at 37°C for 1 h. Protein bands were assessed by Image Lab software (Bio-Rad, USA). ImageJ software was used to measure band intensity.

### 2.9. Statistical Analysis

Data collected were expressed as the mean ± SEM. Comparison between two or more groups was performed using the Student *t*-test and ANOVA for normal variables or the Mann-Whitney *U* test and Kruskal-Wallis test for nonnormal variables that could not be log transformed (e.g., because of frequent zero values). All statistical tests were performed using the SPSS 16.0 software. *P* < 0.05 was considered indicative of a statistically significant difference.

## 3. Results

### 3.1. Generation of hiPSC-CMs

**Figure 1:**
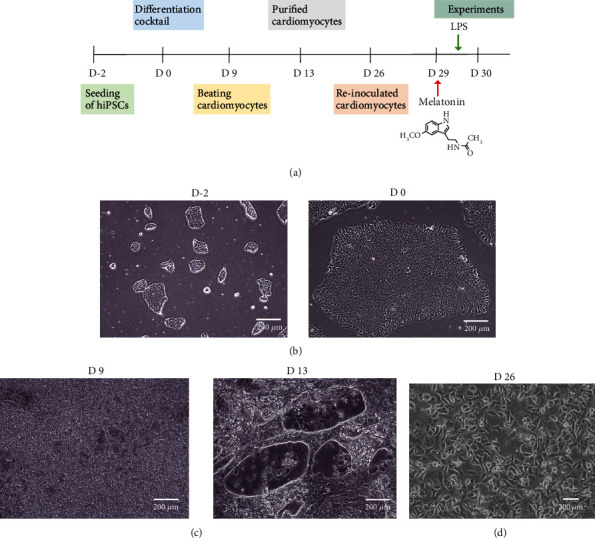
Experimental timeline and cell culture. (a) Flow chart of experiments. (b) Bright-field images of cultured hiPSCs. Scale bars: 200 *μ*m. (c) Bright-field images of beating cardiomyocytes on day 9 and purified cardiomyocytes on day 13. Scale bars: 200 *μ*m. (d) Bright-field images of purified and reinoculated hiPSC-CMs. Scale bars: 200 *μ*m.

**Figure 2:**
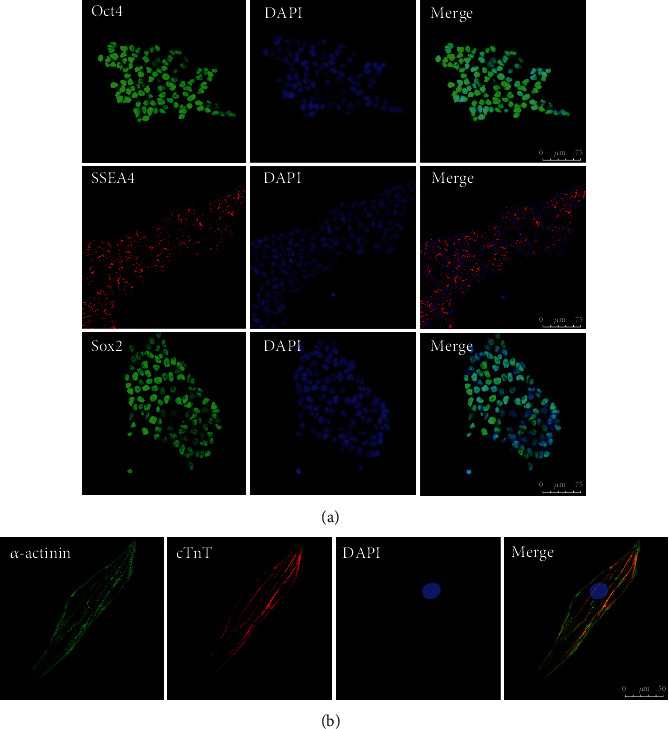
Characterization of hiPSCs and hiPSC-CMs. (a) Immunostaining of hiPSCs before differentiation: Oct4 (green), SSEA4 (red), Sox2(green), and DAPI (blue). (b) Immunostaining of hiPSC-CMs: *α*-actinin (green), cTnT (red), and DAPI (blue). Oct4: octamer-binding transcription factor 4; SSEA4: stage-specific embryonic antigen 4; Sox2: SRY-box transcription factor 2.

A flow chart of the experiment is shown in Figure 1(a). To obtain cardiomyocytes with high differentiation efficiency, hiPSCs were differentiated into cardiomyocytes upon cell confluence reaching 70~80%, using a small-molecule-based monolayer differentiation method (Figure 1(b)). hiPSC-CMs contracted spontaneously on day 8 after induction, and the beating cells were purified on day 14 and then reinoculated in 6-well plates (Figure 1(c)). Immunostaining revealed positive staining of the stem cell markers Oct4, SSEA4, and Sox2, confirming the pluripotency of hiPSCs (Figure 2(a)). Immunostaining also revealed positivity for the cardiomyocyte markers cTnT and *α*-actinin in hiPSC-CMs after purification, demonstrating the success of hiPSC-CM differentiation (Figure 2(b)).

### 3.2. Mel Protected hiPSC-CMs from LPS-Induced Injury

**Figure 3:**
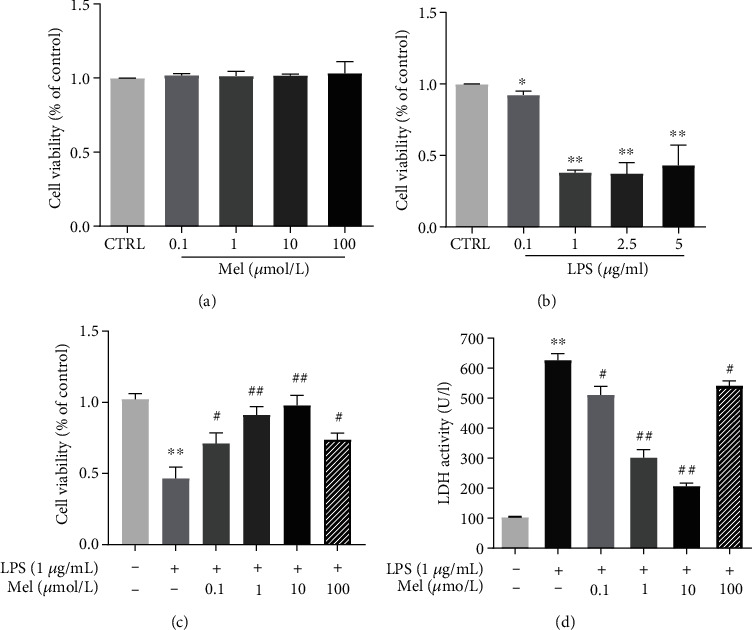
Melatonin improves cell viability in LPS-treated hiPSC-CMs. (a) hiPSC-CMs were challenged with various doses of melatonin (0.1, 1, 10, and 100 *μ*mol/l) for 12 h; the viability of hiPSC-CMs was measured by CCK-8 assay. (b) hiPSC-CMs were treated with various doses of LPS (0.1, 1, 2.5, or 5 *μ*g/ml) for 24 h. (c) hiPSC-CMs were incubated with LPS (1 *μ*g/ml) for 24 h post melatonin pretreatment (0.1, 1, 10, or 100 *μ*mol/l) for 12 h; then, the cell viability of hiPSC-CMs was assessed by CCK-8 assay. (d) Measurement of LDH release level in the same condition of (c). *n* ≥ 3; ^∗^*P* < 0.05 vs. control; ^∗∗^*P* < 0.01 vs. control; ^#^*P* < 0.05 vs. LPS; ^##^*P* < 0.01 vs. LPS.

We then tested the cardioprotective properties of Mel against LPS-induced hiPSC-CM injury. hiPSC-CMs were preconditioned with different concentrations of Mel (0.1, 1, 10, and 100 *μ*mol/l) for 12 hours followed by LPS (0.1, 0.5, 1, 2.5, and 5 *μ*g/ml) treatment for 24 hours, and cell viability was tested by CCK-8. The results showed no significant differences between normal hiPSC-CMs treated with various concentrations of Mel for 12 h (Figure 3(a)), whereas LPS treatment dose-dependently reduced cell viability; the decrease was most evident at a dose of 1 *μ*g/ml for 24 h (Figure 3(b)). However, Mel effectively reversed the LPS-induced decline in hiPSC-CM viability (Figure 3(c)). To determine the ability of Mel to attenuate LPS-induced injuries in hiPSC-CMs, lactate dehydrogenase (LDH) activity was measured in cells from different groups. LDH activity was obviously higher in the LPS group than that in the CTRL group; however, Mel effectively attenuated LPS-induced LDH activity upregulation in a dose-dependent manner (Figure 3(d)).

### 3.3. Mel Alleviated LPS-Induced Pyroptotic Cell Death in hiPSC-CMs

**Figure 4:**
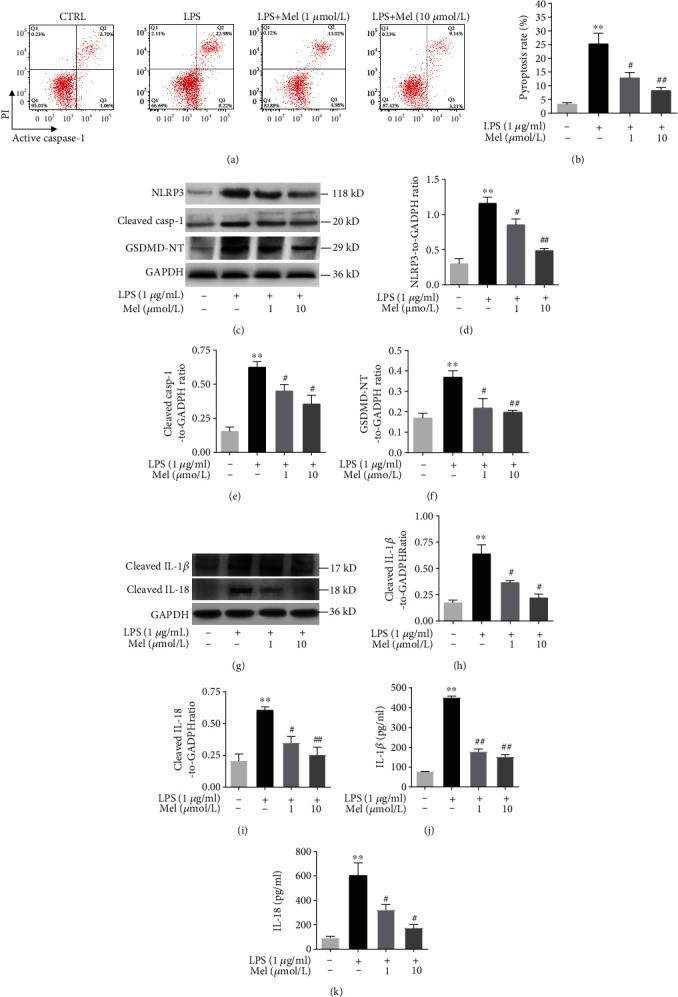
Melatonin reduced cell pyroptosis in LPS-exposed hiPSC-CMs. (a) Representative images of flow cytometry results. (b) Quantitative analysis of pyroptosis rate. (c) Representative immunoblots of NLRP3, cleaved casp-1, and GSDMD-NT. (d) Quantitative analysis of NLRP3. (e) Quantitative analysis of cleaved casp-1. (f) Quantitative analysis of GSDMD-NT. (g) Representative immunoblots of cleaved IL-1*β* and cleaved IL-18. (h) Quantitative analysis of cleaved IL-1*β*. (i) Quantitative analysis of cleaved IL-18. (j) IL-1*β* level examined by ELISA assay. (k) IL-18 level examined by ELISA assay. *n* ≥ 3; ^∗^*P* < 0.05 vs. control; ^∗∗^*P* < 0.01 vs. control; ^#^*P* < 0.05 vs. LPS; ^##^*P* < 0.01 vs. LPS.

We next examined the effects of Mel and LPS on the pyroptotic cell death of hiPSC-CMs. The results showed that LPS significantly increased PI and active caspase-1 (p20) double-positive pyroptotic cells compared with the CTRL group. However, Mel dose-dependently alleviated the pyroptotic cell death rate (Figures 4(a) and 4(b)). The Western blot analysis validated LPS-induced hiPSC-CM death as indeed pyroptosis, as evidenced by the increased protein expression of NLRP3 and enhanced cleavage of caspase-1 and GSDMD-NT compared with the CTRL group; however, Mel reduced these expression levels dose-dependently (Figures 4(c)–4(f)). Cleaved IL-1*β* and cleaved IL-18 are two important indicators of NLRP3 inflammasome activation. We measured their expressions by Western blotting. The results showed that while LPS significantly increased the expression of cleaved IL-1*β* and cleaved IL-18 compared with the CTRL group, Mel reduced their levels dose-dependently (Figures 4(g) and 4(i)). We further measured the concentrations of IL-1*β* and IL-18 in the cell supernatants. While IL-1*β* and IL-18 expression was significantly upregulated in the LPS treatment group compared with the CTRL group, Mel supplementation decreased these increases in a dose-dependent fashion (Figures 4(j) and 4(k)).

### 3.4. Mel Promoted Cell Autophagy in LPS-Treated hiPSC-CMs

**Figure 5:**
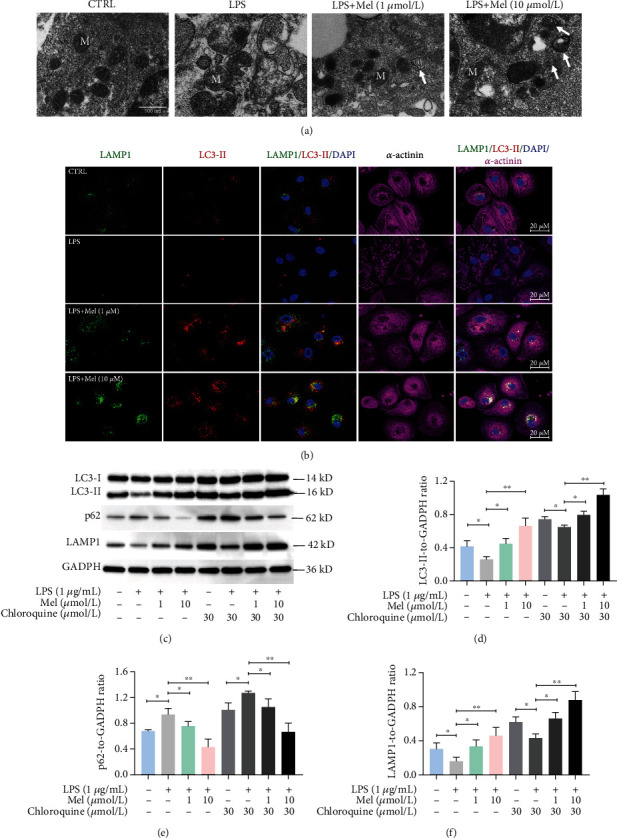
Melatonin promotes autophagy in LPS-exposed hiPSC-CMs. (a) Transmission electron microscope images of hiPSC-CMs in LPS+MEL (1 *μ*mol/l) and LPS+MEL (10 *μ*mol/l) cells. M indicates representative mitochondria. Arrows indicate autophagosomes. Scale bar: 500 nm. (b) Representative immunofluorescent images for LAMP1 (green), LC3-II (red), *α*-actinin (purple), and DAPI (blue). Scale bar: 20 *μ*m. (c) Representative immunoblots of LC3-II, p62, and LAMP1. (d) Quantitative analysis of LC3-II. (e) Quantitative analysis of p62. (f) Quantitative analysis of LAMP1. *n* ≥ 3; ^∗^*P* < 0.05; ^∗∗^*P* < 0.01.

As autophagy is a vital regulator in maintaining intracellular stability, we further explored the involvement of autophagy in Mel-elicited cytoprotective effects. We first performed transmission electron microscopy (TEM) to evaluate the ultrastructure of the treated hiPSC-CMs. TEM showed normal mitochondrial morphology with clear mitochondrial cristae of hiPSC-CMs in the CTRL group, while LPS induced swollen mitochondria with lysis of the cristae, indicating damage to mitochondrial structures. Double-membrane autophagosomes (an indicator of the presence of autophagy) were observed in the LPS+MEL group (both 1 *μ*mol/l and 10 *μ*mol/l Mel), indicating that Mel induced autophagy in LPS-treated hiPSC-CMs (Figure 5(a)). Autophagosome formation and degradation are vital steps in autophagy. We also monitored autophagy by immunofluorescence staining of LC3-II, LAMP1 (lysosomal-associated membrane protein 1), and *α*-actinin (cardiomyocyte marker) in treated hiPSC-CMs. Compared with the CTRL group, hiPSC-CMs treated with LPS exhibited much fewer acidic vesicles (revealed by LAMP1 staining) and fewer autophagosomes (revealed by LC3-II staining); however, Mel dose-dependently reversed this phenomenon (Figure 5(b)). To validate the TEM and staining findings, we performed Western blotting-based detection of the autophagy and lysosomal markers LC3-II, p62, and LAMP1. Mel dose-dependently increased LC3-II and LAMP1 but abolished LPS-induced p62 upregulation. To better understand the effect of Mel on promoting autophagy, we employed chloroquine, a drug that blocks the fusion of autophagic vesicles and lysosomes, to analyze the autophagic flux in each group. When cells were cotreated with chloroquine, significant accumulation of LC3-II, p62, and LAMP1 protein was observed in all groups, suggesting the successful blockade of autophagic flux. Furthermore, a similar trend in the changes in LC3-II, p62, and LAMP1 protein expressions was observed upon chloroquine treatment in the LPS group and Mel groups compared with no chloroquine treatment (Figures 5(c)–5(f)). Collectively, these results demonstrated that Mel indeed enhanced autophagic flux in LPS-injured hiPSC-CMs and that this effect might be involved in Mel-elicited cytoprotective effects.

### 3.5. Mel Ameliorated Pyroptosis in LPS-Exposed hiPSC-CMs by Activating Autophagy

**Figure 6:**
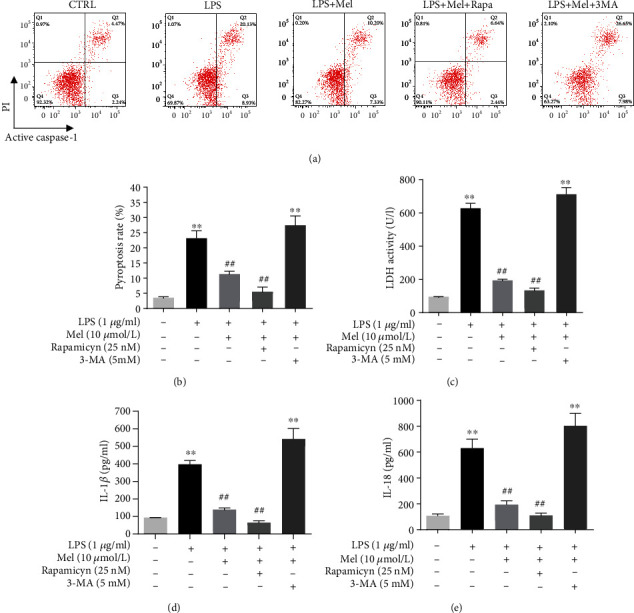
Melatonin attenuates pyroptosis in LPS-exposed hiPSC-CMs *via* enhancing autophagy. (a) Representative images of flow cytometry results. (b) Quantitative analysis of pyroptosis rate. (c) Measurement of LDH release level. (d) Supernatant IL-1*β* level examined by the ELISA assay. (e) Supernatant IL-18 level examined by the ELISA assay. Mel was administered at 10 *μ*mol/l. *n* ≥ 3; ^∗^*P* < 0.05 vs. control; ^∗∗^*P* < 0.01 vs. control; ^#^*P* < 0.05 vs. LPS; ^##^*P* < 0.01 vs. LPS.

We next employed rapamycin (an autophagy activator) and 3-MA (an autophagy inhibitor) to examine the role of Mel in restoring autophagy and its antipyroptotic actions. Flow cytometry analyses revealed that LPS significantly increased the number of PI and active caspase-1 double-stained pyroptotic cells compared with those in the CTRL group. Mel markedly reduced LPS-induced pyroptotic cell death rates, while rapamycin treatment further decreased pyroptotic cell death rates compared with Mel alone (Figures 6(a) and 6(b)). In addition, the combination of Mel and rapamycin considerably increased LDH activity compared to the effect of other treatments (Figure 6(c)). We further measured the expression of IL-1*β* and IL-18 cells from each group. LPS treatment significantly increased the secretion of IL-1*β* and IL-18 compared with that of the CTRL group. However, Mel markedly decreased the levels of IL-1*β* and IL-18 in the LPS-treated groups, with 3-MA reversing its impact and increasing their levels. Combined Mel and rapamycin further drastically decreased IL-1*β* and IL-18 secretion compared with any other group (Figures 6(d) and 6(e)). These results demonstrated that Mel attenuated pyroptosis in LPS-exposed hiPSC-CMs by enhancing autophagy.

In this study, we established a novel hiPSC-CM model to mimic the development of septic cardiomyopathy *in vitro* and used it initially to assess Mel's ability to alleviate LPS-induced hiPSC-CM injuries. For the first time, we demonstrated that Mel alleviated the pyroptosis of hiPSC-CMs exposed to LPS by activating cell autophagy.

## 4. Discussion

Mel is a neuroendocrine hormone produced mainly by the pineal gland, and it is a pleiotropic molecule; it acts as a free radical scavenger, antioxidant, and regulator of circadian rhythm in mammals [26]. Mel also has anti-inflammatory properties and controls glucose and lipid metabolism [27], and there is evidence that it plays a key role in the pathophysiology of multiple cardiovascular diseases, including hyperlipidemia [28], diabetic cardiomyopathy [17], atherosclerosis [20], and myocardial ischemia-reperfusion injury [19]. Supposedly, Mel protects against organ dysfunction, improves survival rates in animal models of sepsis [29, 30], and safeguards against sepsis-induced cardiac dysfunction [31]. In line with these findings, we demonstrated that treatment with Mel (1 or 10 *μ*mol/l) significantly increased hiPSC-CM viability and considerably decreased LDH release induced by 1 *μ*g/ml LPS, indicating that Mel attenuated cardiomyocyte injuries in the *in vitro* sepsis model. However, the underlying mechanisms of these actions remain to be further elucidated.

Pyroptosis is associated with NLRP3 inflammasome activation and is characterized as inflammatory caspase- (mainly caspase-1-) dependent programmed cell death. After activation of the NLRP3 inflammasome, caspase-1-cleaved GSDMD N-terminal (GSDMD-NT) initiates pyroptosis, which may instigate and expand inflammation, resulting in LPS-injured hiPSC-CMs [7]. Recent investigations have shown that cardiomyocyte pyroptosis occurs in a septic heart [10, 32]. Nevertheless, we had to distinguish pyroptotic cells from apoptotic cells; therefore, we evaluated propidium (PI) and active caspase-1 double-positive cardiomyocytes using flow cytometry following a previously established protocol to evaluate cell pyroptosis [33]. The cell pyroptosis rate in the LPS treatment group was significantly upregulated. However, pretreating CMs with 1 *μ*mol/l or 10 *μ*mol/l Mel for 12 h significantly reduced LPS-induced cell pyroptosis, proving that Mel could lessen LPS-induced pyroptotic hiPSC-CM deaths. Here, our data support the notion that the NLRP3 inflammasome is activated by LPS in cardiomyocytes to mediate pyroptosis for sepsis pathogenicity. However, Mel pretreatment markedly diminished pyroptosis-related molecular levels of NLRP3, cleaved caspase-1, and GSDMD-NT compared with the LPS treatment group. Our findings suggested that Mel could alleviate LPS-induced pyroptotic hiPSC-CM death. Inflammatory impairments are the core clinical symptoms of sepsis that progressively exacerbate organ infections and injuries; therefore, their alleviation must be an urgent matter to consider when developing new therapeutic approaches for sepsis. As mentioned above, NLRP3 inflammasomes activate the protease caspase-1, which cleaves IL-1*β* and IL-18 to generate the corresponding mature cytokines and controls their secretion to form pyroptosis. We revealed here that Mel supplementation reduced cleaved IL-1*β* and cleaved IL-18 expression dose-dependently in LPS-treated hiPSC-CMs, a finding confirmed by the dose-dependent lower levels of proinflammatory factors (IL-1*β* and IL-18) in the Mel supplementation groups.

Properly regulated levels of autophagy are vital for cellular homeostasis in the course of various inflammatory diseases [34–36]. Supposedly, Mel enhances autophagy in sepsis-induced hearts and abrogates cardiac dysfunction and apoptosis [37], and there is also evidence that the overexpression of Beclin-1, an important autophagy initiator, alleviates LPS-induced inflammation and cardiac dysfunction [38]; if so, then Mel-mediated protection in a septic heart results from the activation of autophagy, suggesting that the removal of damaged and dysfunctional mitochondria could be the mechanism by which Mel safeguards against sepsis. Accordingly, in the present study, Mel reduced the number of intracellular swollen and damaged mitochondria with lysis of the cristae and increased the number of double-membrane structures resembling autophagosomes in LPS-exposed hiPSC-CMs. We also characterized autophagy *via* autophagy-related proteins in the LPS+MEL groups, with Mel increasing LC3-II expression but decreasing p62 levels. These findings indicate that Mel could induce protective autophagy in LPS-treated hiPSC-CMs.

The interplay of pyroptosis and autophagy is thought to be fundamental to maintaining cellular homeostasis in the course of various inflammatory diseases. A recent report showed that autophagy activation exerts a neuroprotective effect by inhibiting pyroptosis in a murine model of traumatic brain injury [39]. In another recent study, quercetin, a natural flavonoid, reportedly prevented neuronal injury *via* inhibition of mtROS-mediated NLRP3 inflammasome activation in microglia by promoting mitophagy [40]. Likewise, our results suggested that rapamycin, a drug that fosters autophagy, acted like Mel to decrease LPS-induced pyroptosis. Moreover, combined Mel and rapamycin further significantly reduced cell death rates. However, coincubation with the autophagy inhibitor 3-MA abolished the Mel-elicited antipyroptotic effects. These results indicate that Mel attenuates pyroptosis in LPS-exposed hiPSC-CMs by enhancing autophagic activity.

## 5. Conclusion

Our current study has several limitations. First and foremost, we failed to validate our major findings in animals and patients; thus, whether Mel protects against endotoxemia-induced cardiac pyroptosis via activation of autophagy in vivo remains to be defined. Second, although our data supported the efficacy of Mel in alleviating LPS-induced pyroptotic cardiomyocyte death by activating autophagy, the exact underlying molecular mechanisms of this action were not identified in the present study. Third, the hiPSC-CMs used in this study are still fetal-like and immature and cannot fully recapitulate the phenotypes of human adult cardiomyocytes. Finally, endotoxemia in sepsis is a systematic response that involves many other tissues and organs apart from the heart, and the antipyroptotic action of Mel in other septic tissues and organs remains to be clarified in future studies. Nonetheless, our study is the first to report the antipyroptotic action of Mel in LPS-injured hiPSC-CMs, and it may provide preliminary data for the clinical application of Mel in sepsis and other pyroptosis-related inflammatory diseases.

In summary, we revealed for the first time that Mel exerts an antipyroptotic function in LPS-induced hiPSC-CM injuries by activating autophagy, which could be crucial in establishing the mechanism of Mel-mediated cytoprotection in septic heart injury. Therefore, our study provides preliminary data for employing Mel as a promising agent for retarding pyroptosis in various inflammatory diseases.

## Data Availability

The data used to support the findings of this study are available from the corresponding author upon request.

## Supplementary Materials

Supplementary Materials Supplementary Figure 1 (Figure S1): Mel pretreatment (10 *μ*mol/l) in LPS-injured hiPSC-CMs for 6 h, 12 h, 18 h, and 24 h. (a) Measurement of the cell viability of hiPSC-CMs by CCK-8 assay. (b) Measurement of LDH release level.
