# Supplementary material for: Melatonin Alleviates LPS-Induced Pyroptotic Cell Death in Human Stem Cell-Derived Cardiomyocytes by Activating Autophagy
Source: Stem Cells Int. 2021 Nov 27;2021:8120403. doi: 10.1155/2021/8120403 (PMC8643260; doi:10.1155/2021/8120403)
Supplement: Supplementary Materials — Supplementary Figure 1 (Figure S1): Mel pretreatment (10 μmol/l) in LPS-injured hiPSC-CMs for 6 h, 12 h, 18 h, and 24 h. (a) Measurement of the cell viability of hiPSC-CMs by CCK-8 assay. (b) Measurement of LDH release level. [file 8120403.f1.docx]

**
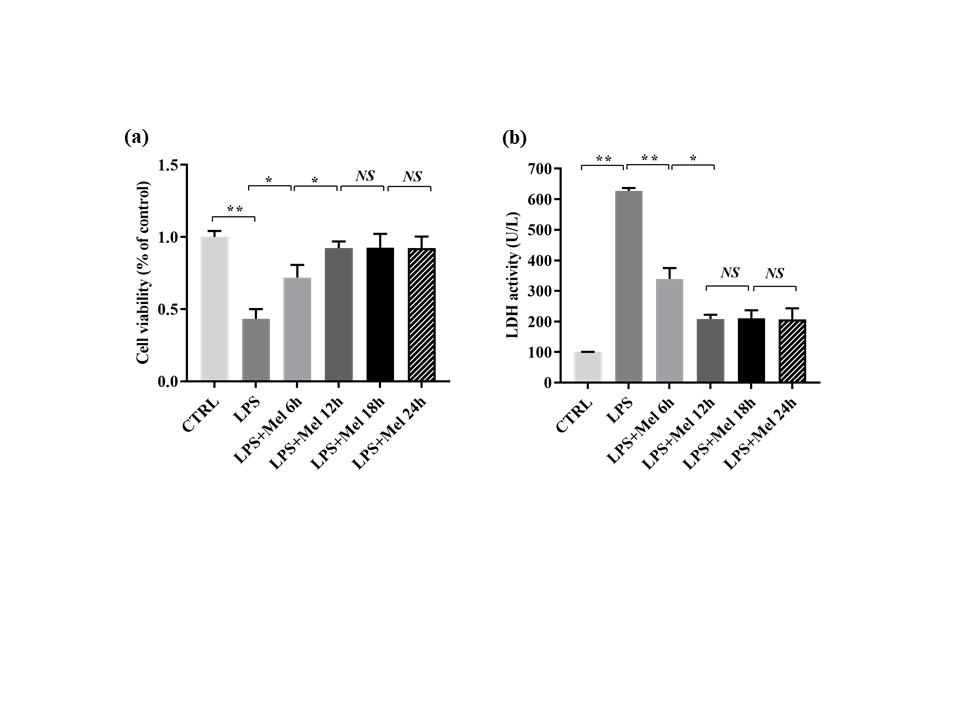
**

**Figure S1.** **Mel pretreatment (10 μmol/L) in LPS-injured hiPSC-CMs for 6h, 12h, 18h and 24h.** (a) Measurement of the cell viability of hiPSC‐CMs by CCK‐8 assay. (b) Measurement of LDH release level. n≥3; * *P*<0.05, ** *P*<0.01, *NS*: no significant difference.
